# Bibliometric analysis of resilience in stroke from 2000 to 2024 using CiteSpace and VOSviewer

**DOI:** 10.3389/fpsyg.2025.1452249

**Published:** 2025-05-30

**Authors:** Yaoyao Li, Kim Lam Soh, XiuJuan Jing, Lili Wei, Ruthpackiavathy Rajen Durai, Kim Geok Soh

**Affiliations:** ^1^Department of Nursing, Faculty of Medicine and Health Sciences, Universiti Putra Malaysia, Serdang, Malaysia; ^2^Department of Neurology, Zibo Central Hospital, Zibo, Shandong, China; ^3^Department of Dean’s Office, The Affiliated Hospital of Qingdao University, Qingdao, Shandong, China; ^4^Department of Sports Studies, Faculty of Educational Studies, Universiti Putra Malaysia, Serdang, Malaysia

**Keywords:** bibliometric, stroke, resilience, CiteSpace, VOSviewer, mental health

## Abstract

**Objective:**

This study aims to conduct a bibliometric analysis of research literature on psychological resilience among stroke survivors published from 2000 to 2024, utilizing VOSviewer and CiteSpace.

**Methods:**

The literature data was sourced from the Web of Science Core Collection database (WoSCC). A total of 424 relevant articles, published between January 1, 2000, and April 30, 2024, were included. To analyze the literature, the software tools CiteSpace and VOSviewer were employed, examining perspectives such as authorship, country of origin, institutions, journals, references, and keywords.

**Results:**

Since 2015, the annual publication output has steadily increased, reaching a peak in 2022 (65 articles). The United States is the most prolific contributor, with Harvard University being the leading institution in this field. Zhang W and Vranceanu A have emerged as the authors with the highest productivity, each boasting five published articles. “Stroke” is the most co-cited journal (204 times) with a high impact factor (IF 2022, 8.4). The most frequently occurring keywords are “stroke,” “resilience,” “depression,” “health,” and “quality of life.” Emerging trends include research on post-stroke cognitive impairment, meta-analyses, population differences, guideline development, and symptom management.

**Conclusion:**

This bibliometric study indicates the increased scholarly interest in investigating psychological resilience among persons who have survived a stroke over the last 24 years. The United States and China have emerged as the leading contributors to this study area, with international collaboration on the rise. To enhance this subject, subsequent studies should target refining theoretical frameworks, enhancing assessment instruments, establishing evidence-based guidelines, and developing tailored therapies that increase psychological resilience and holistic well-being for stroke survivors.

## 1 Introduction

Stroke, a severe cerebrovascular disorder arising from the cessation of blood supply to the brain, can cause regional neural tissue injury and a spectrum of neurological abnormalities. The Global Burden of Disease research rates stroke as the second most widespread cause of mortality globally (Sirdani et al., 2021; Xiong et al., 2022). Nearly 50% of stroke survivors face varying levels of disability, which can manifest as deficits in motor control, sensory perception, speech, and cognitive function, significantly impairing their capacity to carry out everyday tasks and reducing their overall quality of life (Nikolovska and Taci, 2023; Fan and Lei, 2022). Beyond physical restrictions, stroke survivors frequently battle with deep psychological issues, such as depression, anxiety, panic disorder, and post-traumatic stress disorder (Gugliandolo et al., 2021; Ohashi et al., 2023). These unfavorable emotional states not only aggravate patients’ functional limitations and disability but also emerge as crucial determinants influencing stroke recovery (Stuckey et al., 2021; Xiong et al., 2022). Consequently, stroke rehabilitation should focus not only on the recovery of physical function but also on the maintenance and promotion of patients’ mental health (Nikolovska and Taci, 2023; Fan and Lei, 2022).

Psychological resilience, defined as an individual’s capacity to maintain positive adaptation and sustain physical and mental health when confronted with adversity, plays an important role in the rehabilitation of stroke survivors. Studies have demonstrated that stroke patients possessing high levels of psychological resilience exhibit a greater ability to accept the reality of their condition, adapt their mindset, and actively participate in rehabilitation training, ultimately leading to superior functional outcomes in daily living activities and social engagement (Han et al., 2021; Heltty and Zahalim, 2023). Moreover, psychological resilience serves as a protective factor against emotional disorders commonly experienced by stroke survivors, such as depression and anxiety, thereby promoting overall quality of life and well-being (Tsai et al., 2023; Norvang et al., 2022). Considering the vital importance of psychological resilience in stroke rehabilitation, there is a pressing need to intensify research efforts in this area (Yan and Lin, 2022; Zhang et al., 2022).

Traditional reviews focus on the elaboration of article content and struggle to reveal hot topics in research themes and collaboration among scholars. Bibliometric analysis, a method that applies mathematical and statistical techniques, is used to gather and analyze literature data such as authors, countries, institutions, keywords, cited authors, and cited journals through specialized software. This approach employs quantitative methods to uncover the macro-level landscape of a field (Donthu et al., 2021), providing insights into the development trends of a discipline (Muraleedharan and Chandak, 2023). Bibliometric analysis, a commonly employed technique in various disciplines including biomedicine, social sciences, engineering, technology, and management, enables researchers to identify potential collaborators in the field of stroke psychological resilience research. This approach enables academics to understand the history of research focal points and actively assess the developing landscape of the field. By utilizing this complete approach, scholars can acquire a thorough comprehension of the current state of research on psychological resilience in stroke survivors and predict future investigation paths (Chen et al., 2023; Bin Suliman et al., 2023).

In recent years, there has been a rising interest in the literature concerning psychological resilience among stroke survivors. This indicates that researchers are increasingly concentrating on the relationship between post-stroke psychological resilience and rehabilitation outcomes. Despite this growing interest, no bibliometric studies have been specifically conducted on psychological resilience among stroke survivors. Given the importance of psychological resilience in stroke rehabilitation, it is essential to utilize bibliometric methods to thoroughly analyze the development context of relevant national and global research. This approach will help researchers identify research hotspots, foundational knowledge, and frontier dynamics within the field, ultimately providing direction and guidance for future, in-depth studies of psychological resilience among stroke survivors.

## 2 Materials and methods

### 2.1 Search strategy

This study relied on the Web of Science Core Collection (WoSCC) to retrieve relevant literature. As a widely recognized high-quality digital literature database, WoSCC is frequently considered the optimal resource for conducting bibliometric analyses (Yan and Zhiping, 2023). The Science Citation Index (SCI) Expanded (1900-present) was selected as the retrieval citation index to guarantee the thoroughness and accuracy of the collected data (Liu et al., 2021).

The search strategy was as follows: TS = (“stroke*” OR “cerebrovascular accident*” OR “cerebrovascular disorder*” OR “cerebrovascular disease*” OR “brain vascular accident*” OR “brain ischemia*” OR “cerebral infarction*” OR “intracranial hemorrhage*”) AND TS = (“resilience” OR “psychological resilience” OR “psychosocial resilience” OR “emotional resilience” OR “mental resilience” OR “post-stroke resilience”). The search time range was set from January 1, 2000, to April 30, 2024, to cover the latest advances in stroke psychological resilience research since the beginning of the 21st century. The main types of literature included in the analysis were research articles and review articles, to comprehensively reflect the original research results and summary views in the field. Considering that English is the common language in international research, we limited the language of the literature to English to ensure the readability of the included literature and the universality of the research results.

After initial retrieval and screening, we finally included 424 articles for subsequent bibliometric analysis.

### 2.2 Analysis tools

To comprehensively analyze the bibliometric characteristics of the literature of literature related to stroke and psychological resilience, this study employed a variety of bibliometric analysis tools and visualization software. First, we used Microsoft Excel 2019 for preliminary statistics and analysis of the raw literature data, including basic characteristics such as the annual distribution of published literature and the composition of literature types.

Next, we conducted a more in-depth bibliometric analysis and visualization using CiteSpace 6.2.3 R3 and VOSviewer 1.6.17 software. CiteSpace is a Java-based information visualization software and a visualization analysis tool capable of exploring the knowledge potential in scientific literature (Markscheffel and Schröter, 2021; Chen et al., 2023). VOSviewer is also a Java-based visualization software developed by Leiden University in the Netherlands, primarily used for constructing and viewing bibliometric knowledge maps. Both software packages are widely applied in bibliometric research (Markscheffel and Schröter, 2021; Chen et al., 2023).

Using these tools, we performed comprehensive bibliometric analyses of the literature on resilience in stroke survivors from multiple perspectives, including author collaboration networks, country distribution characteristics, institutional collaboration patterns, journal citation patterns, and the structure and distribution of research topics and hotspots. This provided data support for understanding the developmental trajectory and future trends in this field.

### 2.3 Research ethics

This study was conducted as a bibliometric analysis. All data were sourced from online resources, and no animals or human subjects were involved. Therefore, ethical approval from an ethics committee was not required.

## 3 Results

### 3.1 Analysis of publication output and citations

**FIGURE 1:**
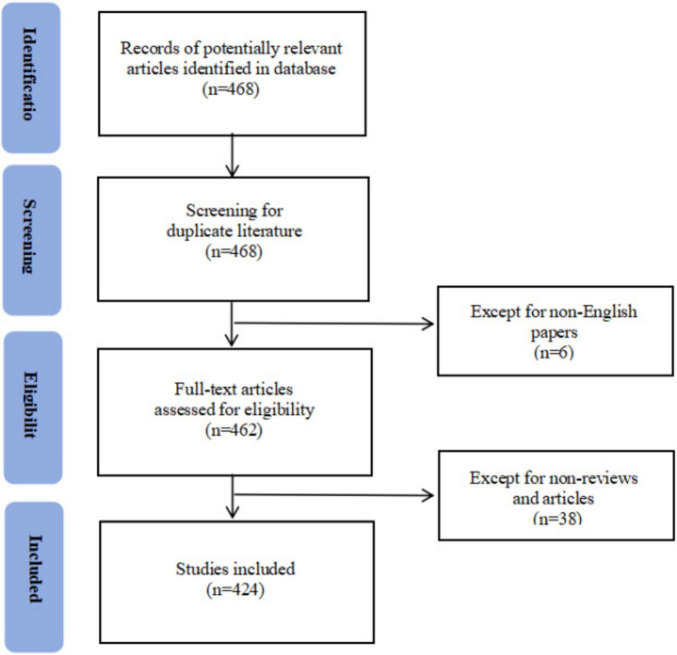
The flow chart of screening process.

**FIGURE 2:**
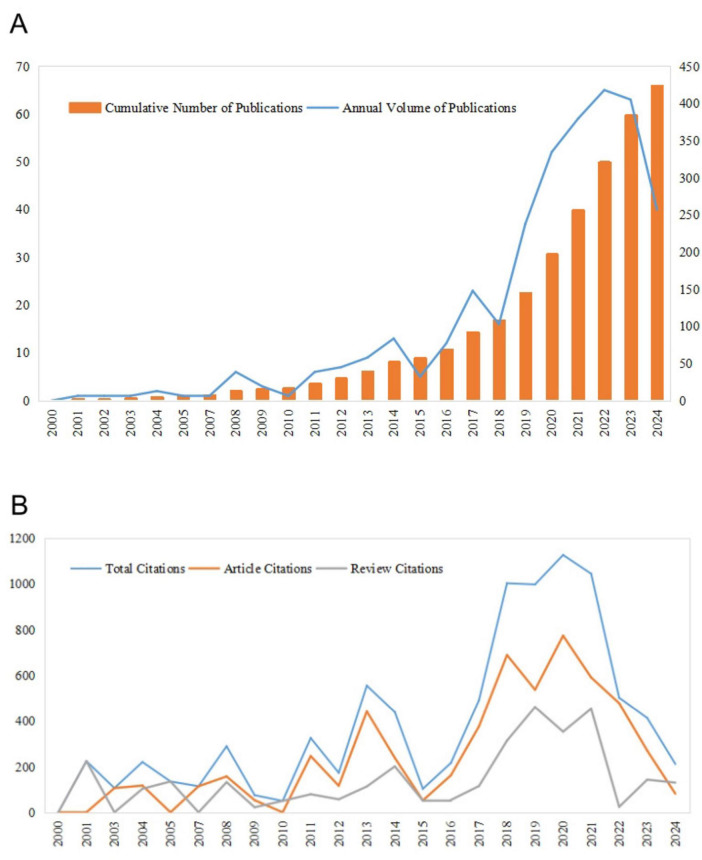
The quantity of publications and citations. **(A)** The publication output and its growth trend from 2000 to 2024. **(B)** The annual citation count from 2000 to 2024.

From January 1, 2000, to April 30, 2024, the Web of Science Core Collection database yielded a total of 424 articles that satisfied the inclusion criteria, comprising 344 research articles and 80 review articles (Figure 1). Figure 2A shows the distribution of annual publications. The results indicate that 2022 was the year with the highest publication output, reaching 65 articles. Overall, the field’s annual scholarly output has shown a steady increasing trend since 2015. These 424 articles have been cited a total of 8,815 times, with 2021 recording the highest number of citations at 1,045 (Figure 2B). Most publications have appeared within the past 5 years, indicating that research on resilience in stroke survivors is a rapidly evolving field that is attracting increasing attention from researchers.

### 3.2 Analysis of authors and co-cited authors

**FIGURE 3:**
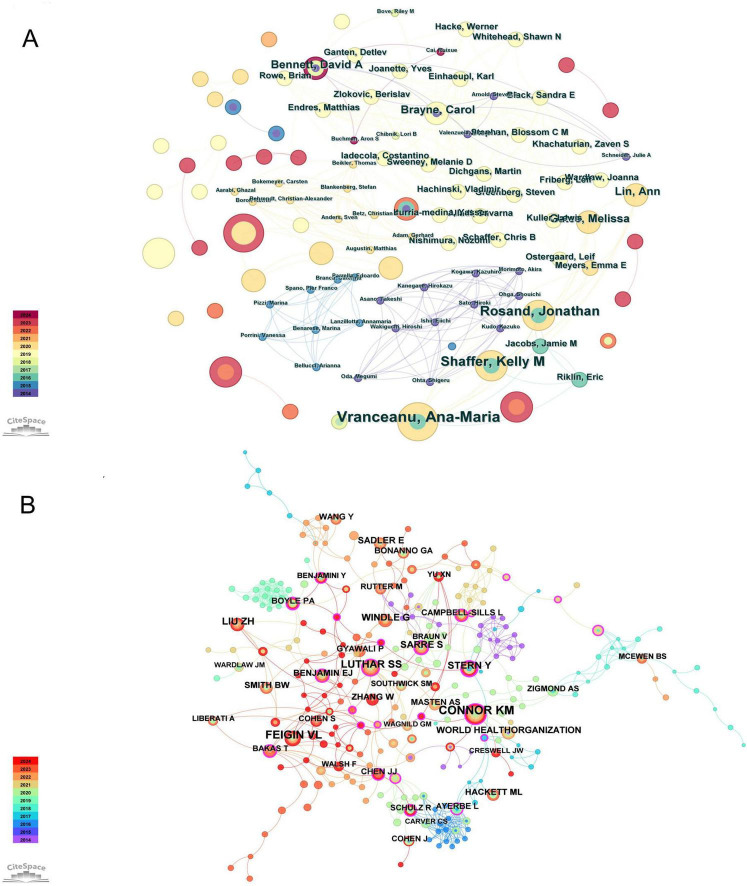
**(A)** Map of authors with publications. **(B)** Map of co-cited authors with publications.

The author collaboration network generated using CiteSpace (Figure 3A) shows that a total of 1,582 authors contributed to the publication of 424 articles. Node size represents the publication volume of authors, while connections indicate collaborative relationships. Table 1 lists the top five authors ranked by publication count, with Zhang W and Vranceanu A tied for first place with five publications, followed by Donlon A, Rosand J, and Shaffer K (four articles each). Meanwhile, the co-cited author network analysis (Figure 3B) reveals that Connor KM (33 citations) is the most frequently cited author, followed by Feigin VL (27 citations), Luthar SS (21 citations), Liu ZH (17 citations), and Windle G (14 citations). Among co-cited authors, Boyle PA demonstrated the highest centrality (0.21). The research contributions of these authors and highly cited researchers collectively constitute the core knowledge foundation for resilience research in stroke survivors.

**TABLE 1:** Top five active authors and co-cited authors.

Rank	Authors	Count	Cited authors	Count times	Cited authors	Centrality
1	Zhang W	5	Connor KM	33	Boyle PA	0.21
2	Vranceanu A	5	Feigin VL	27	Benjamini Y	0.16
3	Donlon A	4	Luthar SS	21	Ayerbe L	0.15
4	Rosand J	4	Liu ZH	17	Stern Y	0.14
5	Shaffer K	4	Windle G	14	Mcewen BS	0.12

### 3.3 Analysis of countries and institutions

**FIGURE 4:**
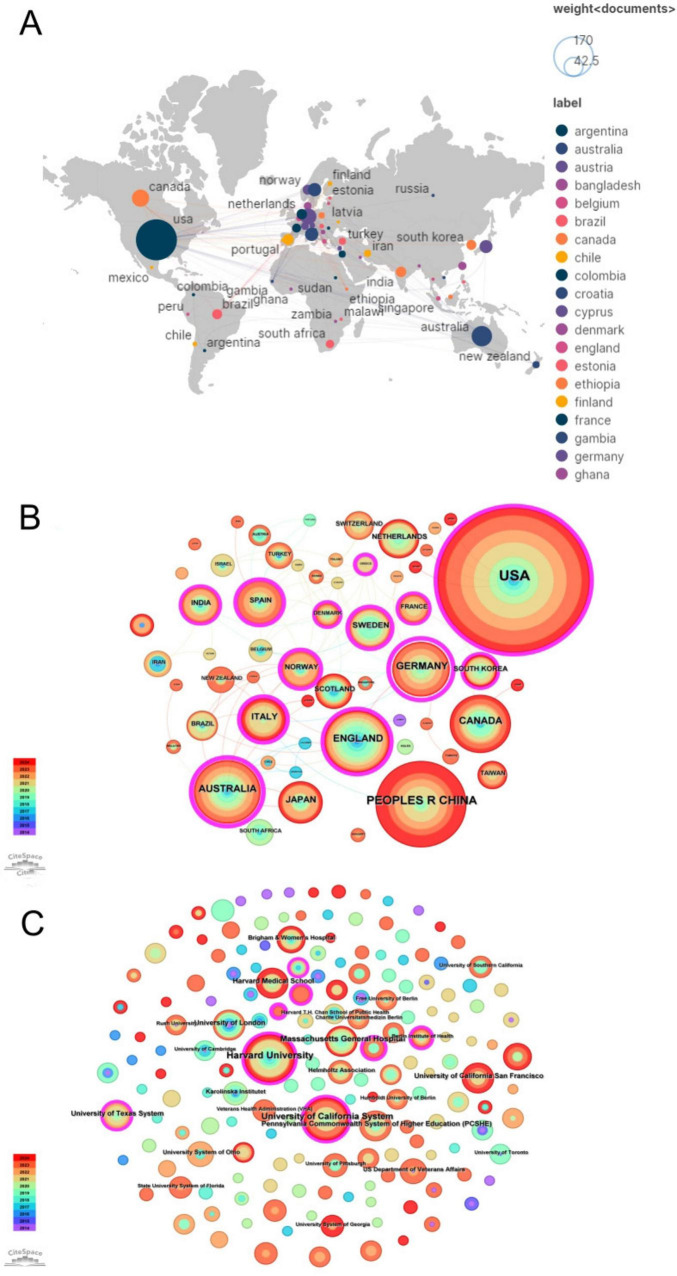
**(A)** The distribution of countries/region. **(B)** Map of countries. **(C)** Map of institutions.

As shown in Figure 4A, publications in this research field originated from 58 countries/regions. The top five countries contributed over 72% of the publications, with the United States (37.03%, 157/424), China (14.39%, 61/424), and the United Kingdom (7.78%, 33/424) ranking as the top three. We used CiteSpace to analyze all articles published between 2000 and 2024 to explore the relationships between publications from different countries. As illustrated in Figure 4B, the analysis generated a merged network comprising 58 nodes and multiple connections. The node size represents publication volume, with larger nodes indicating a higher number of publications, while the links signify collaborative relationships between countries. Table 2 lists the top five countries ranked by publication volume and centrality, with the United States leading in both aspects (157 publications, centrality 0.38), demonstrating its important role in this field.

**TABLE 2:** Top five countries in terms of count and centrality.

Rank	Country	Count	Country	Centrality
1	USA	157	USA	0.38
2	Peoples R China	61	Australia	0.31
3	England	33	England	0.26
4	Australia	32	Germany	0.24
5	Canada	27	Spain	0.14

At the institutional level, we selected “institution” as the node to generate an institutional distribution map (Figure 4C), where larger nodes represent higher publication volumes. Table 3 presents the top five most productive institutions in this research field. Harvard University leads with 27 publications, followed by the University of California System with 22 publications, and Massachusetts General Hospital ranking third with 13 publications. Four of the top five institutions are located in the United States, with only the University of London from the United Kingdom, further emphasizing the United States’ leadership in research on psychological resilience among stroke survivors.

**TABLE 3:** Top five institutions in terms of count and centrality.

Rank	Institution	Count	Institution	Centrality
1	Harvard University	27	Harvard University	0.2
2	University of California System	22	University of Texas System	0.07
3	Massachusetts General Hospital	13	University System of Ohio	0.07
4	University of London	12	University of London	0.06
5	Harvard Medical School	11	Cornell University	0.06

### 3.4 Analysis of journals and co-cited journals

**FIGURE 5:**
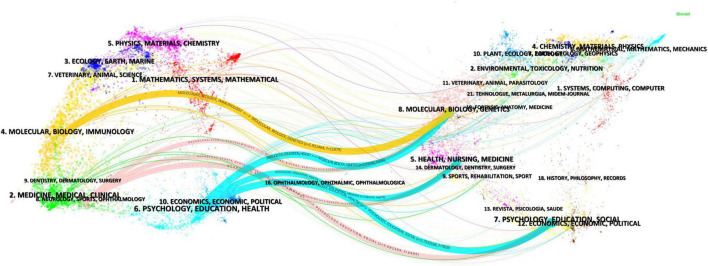
The dual-map overlay illustrating journals and respective publications.

In the field of psychological resilience research among stroke survivors, based on the analysis of 393 journals containing relevant literature. Figure 5 presents the findings of the dual-map analysis of journals. The map on the left illustrates the citing literature, while the map on the right represents the cited literature, with curves denoting citation links. The paths of these links expose the transdisciplinary flow of information within this topic. The *z*-value function amplifies the trajectories with stronger flow; the higher the *z*-value, the thicker the link appears. This study reveals that the literature in the fields of neuroscience, kinesiology, and ophthalmology (indicated by the pink trajectory) was significantly influenced by the publications from psychology, education, and sociology (*z* = 3.08, *f* = 6,489), as well as health sciences, nursing, and medicine (*z* = 1.97, *f* = 4,396). Additionally, the literature in molecular biology, biology, and immunology (represented by the blue trajectory) was impacted by the works in health sciences, nursing, and medicine (*z* = 3.79, *f* = 7,826), while the literature in health sciences, nursing, and medicine (another blue trajectory) was influenced by the literature from psychology, education, and sociology (*z* = 3.22, *f* = 6,741). The literature in the fields of medicine, pharmaceuticals, and clinical practice (yellow trajectory) mainly influenced the literature in the fields of environmental science, toxicology, and nutrition (*z* = 5.95, *f* = 11,879).

Table 4 lists the top 10 journals by publication output. Among them, “Disability and Rehabilitation” ranks first with 9 articles, playing an important role in this field. Additionally, Table 5 lists the five journals with the highest co-cited frequency.

**TABLE 4:** Top 10 academic journals based on publications.

Rank	Source (abbreviations)	Publications	Citations	Average citations/publications	Country	IF (2022)
1	Disability and Rehabilitation (Disabil Rehabil)	9	114	12.67	United Kingdom	2.2
2	BMJ Open	8	96	12	United Kingdom	2.9
3	Stroke	8	245	30.63	United States	8.4
4	Public Library of Science ONE (PLoS ONE)	7	150	21.43	United States	3.7
5	Topics in Stroke Rehabilitation (Top Stroke Rehabil)	7	118	16.86	United Kingdom	2.2
6	Frontiers in Neurology	6	112	18.67	Switzerland	3.4
7	Aphasiology	5	48	9.6	United Kingdom	2.0
8	Neurorehabilitation	5	47	9.4	Netherlands	2.0
9	Scientific Reports	5	38	7.6	United Kingdom	4.6
10	Alzheimer’s and Dementia	4	180	45	United States	14.0

**TABLE 5:** Top five co-cited journals in terms of counts and centrality.

Rank	Co-cited counts	Cited journal (abbreviations)	Centrality	Cited journal (abbreviations)
1	204	Stroke	0.07	Proceedings of the National Academy of Sciences of the United States of America (P Natl Acad Sci USA)
2	155	Public Library of Science ONE (PLoS ONE)	0.07	The American Journal of Psychiatry (Am J Psychiat)
3	120	Proceedings of the National Academy of Sciences of the United States of America (P Natl Acad Sci USA)	0.06	Disability and Rehabilitation (Disabil Rehabil)
4	115	Neurology	0.06	Journal of the American Geriatrics Society (J Am Geriatr Soc)
5	111	The Lancet	0.06	Clinical Rehabilitation (Clin Rehabil)

### 3.5 Analysis of co-cited references

**FIGURE 6:**
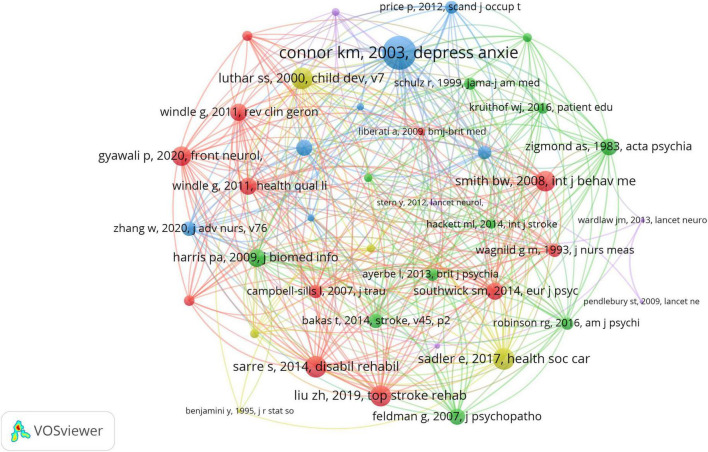
Map of co-cited references with citations.

To investigate the co-citation of references, we used VOSviewer to construct a co-citation network map of references (Figure 6). From 25,269 references, we selected a minimum co-citation frequency threshold of eight, ultimately including 41 references for co-citation analysis. Through this study, we constructed a knowledge network for the field of resilience research in stroke survivors. Table 6 lists the top 10 most frequently co-cited research articles, while Table 7 presents the top 10 most frequently co-cited review articles.

**TABLE 6:** Top 10 co-cited research references in terms of citations.

Rank	Title	Citations	Year	First author	Journal	Document type
1	Development of a new resilience scale: the Connor-Davidson Resilience Scale (CD-RISC)	35	2003	Connor KM	Depression and Anxiety	Research
2	Developing a novel peer support intervention to promote resilience after stroke	18	2016	Sadler E	Health and Social Care in the Community	Research
3	The hospital anxiety and depression scale	16	1983	Zigmond AS and Snaith RP	Acta Psychiatrica Scandinavica	Research
4	Factors associated with quality of life early after ischemic stroke: the role of resilience	15	2019	Liu ZH	Topics in Stroke Rehabilitation	Research
5	The brief resilience scale: assessing the ability to bounce back	14	2008	Smith BW	International Journal of Behavioral Medicine	Research
6	Using thematic analysis in psychology	12	2006	Braun V	Qualitative Research in Psychology	Research
7	Psychometric analysis and refinement of the Connor-Davidson Resilience Scale (CD-RISC): validation of a 10-item measure of resilience	12	2007	Campbell-Sills L	Journal of Traumatic Stress	Research
8	Opposing associations of stress and resilience with functional outcomes in stroke survivors in the chronic phase of stroke: a cross-sectional study	11	2020	Gyawali P	Frontiers in Neurology	Research
9	Evidence for stroke family caregiver and dyad interventions: a statement for healthcare professionals from the American heart association and American stroke association	10	2014	Bakas T	Stroke	Research
10	Factor analysis and psychometric evaluation of the Connor-Davidson Resilience Scale (CD-RISC) with Chinese people	10	2007	Yu XN	Social Behavior and Personality	Research

**TABLE 7:** Top 10 co-cited review references in terms of citations.

Rank	Title	Citations	Year	First author	Journal	Document type
1	The construct of resilience: a critical evaluation and guidelines for future work	18	2003	Luthar SS	Child Development	Review
2	A systematic review of qualitative studies on adjusting after stroke: lessons for the study of resilience	17	2014	Sarre S	Disability and Rehabilitation	Review
3	A methodological review of resilience measurement scales	13	2011	Windle G	Health and Quality of Life Outcomes	Review
4	Natural history, predictors and outcomes of depression after stroke: systematic review and meta-analysis	10	2013	Ayerbe L	The British Journal of Psychiatry	Review
5	Loss, trauma, and human resilience: have we underestimated the human capacity to thrive after extremely aversive events?	9	2004	Bonanno GA.	Psychological Trauma: Theory, Research, Practice, and Policy	Review
6	Resilience definitions, theory, and challenges: interdisciplinary perspectives.	9	2014	Southwick SM	European Journal of Psychotraumatology	Review
7	What is resilience? a review and concept analysis	9	2011	Windle G	Reviews in Clinical Gerontology	Review
8	Frequency of anxiety after stroke: a systematic review and meta-analysis of observational studies	8	2013	Burton CAC	International Journal of Stroke	Review
9	Part I: frequency of depression after stroke: an updated systematic review and meta-analysis of observational studies	8	2014	Hackett ML	International Journal of Stroke	Review
10	Post-stroke depression: a review	8	2016	Robinson RG and Jorge RE	American Journal of Psychiatry	Review

### 3.6 Analysis of keywords

**FIGURE 7:**
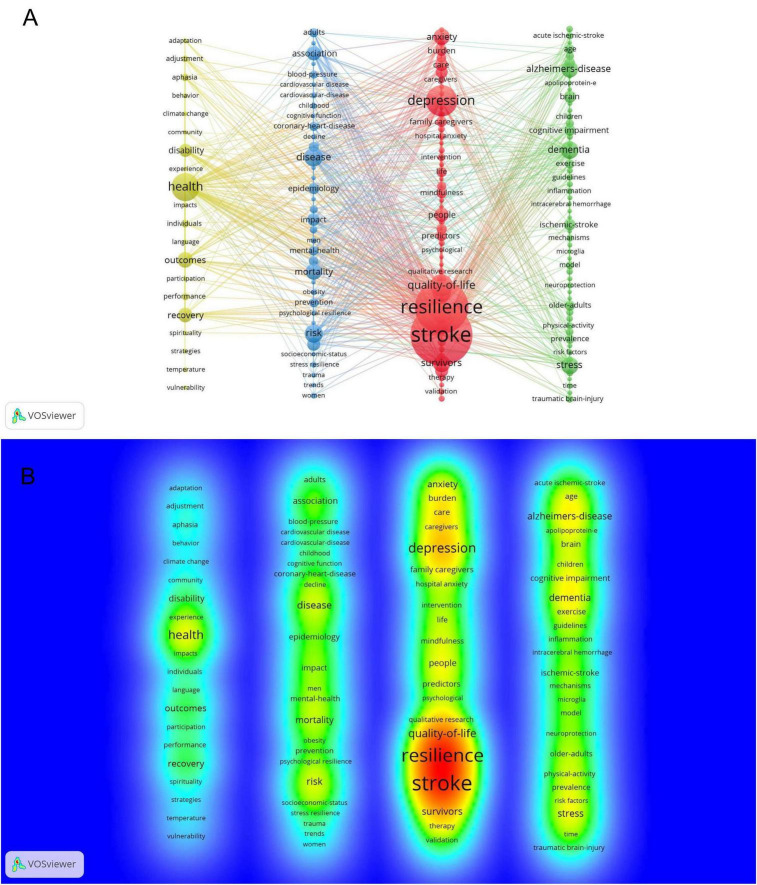
**(A)** Map of co-occurring keywords with occurrences. **(B)** Map of keywords density.

As of April 2024, a total of 2,079 keywords were identified in this research field. Using VOSviewer software, we set a minimum co-occurrence frequency of five, resulting in the inclusion of 165 keywords in the analysis (Figure 7A). In this visualization, nodes represent keywords, node size reflects keyword co-occurrence frequency, and links indicate the strength of associations between keywords, thus constructing a knowledge structure map of the field. Meanwhile, to provide a more intuitive representation of research hotspot, we generated a density visualization map of keywords (Figure 7B), where warmer colors (red areas) indicate higher research focus. Additionally, to reveal the temporal evolution of research hotspots, we conducted the strongest citation bursts of keywords (Table 8), with time intervals represented by blue lines and keyword burst periods indicated by red lines. The analysis results show that the keyword “resilience” had a burst strength of 2.97, with a burst period from 2019 to 2022.

**TABLE 8:** Top 20 keywords with the strongest citation bursts.

Keywords	Year	Strength	Begin	End	2000–2024
Small vessel disease	2014	2.37	2014	2019	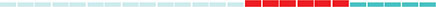
Cell death	2014	1.95	2014	2015	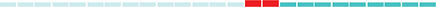
C reactive protein	2014	1.74	2014	2017	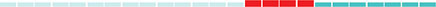
Cognitive impairment	2016	2.57	2016	2019	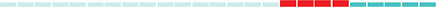
Risk factors	2016	2.49	2016	2017	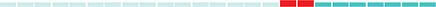
Plasticity	2004	2.04	2017	2019	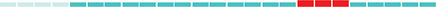
Atrial fibrillation	2017	1.88	2017	2018	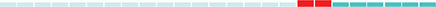
Age	2012	1.73	2018	2019	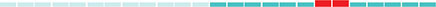
Resilience	2014	2.97	2019	2022	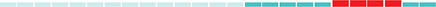
Hospital anxiety	2019	2.79	2019	2020	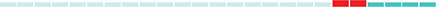
rehabilitation	2019	2.11	2019	2020	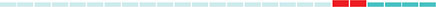
Validation	2019	1.85	2019	2020	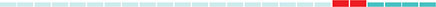
Impact	2020	3.02	2020	2022	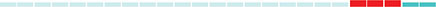
Scale	2020	2.55	2020	2022	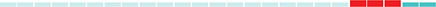
Impairment	2021	2.56	2021	2024	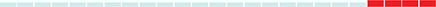
Meta-analysis	2021	2.28	2021	2024	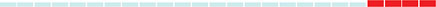
Validity	2021	2.12	2021	2022	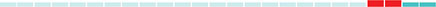
People	2020	3.54	2022	2024	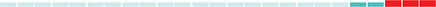
Guidelines	2022	2.92	2022	2024	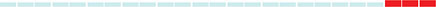
Symptoms	2020	2.19	2022	2024	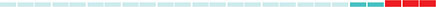

## 4 Discussion

### 4.1 Development trends and overall characteristics of psychological resilience research among stroke survivors

Academic publication volume is an important indicator for measuring the developmental trajectory of a research field (Zafar and Masood, 2020). Figure 2A illustrates the publication trends in psychological resilience among stroke survivors from 2000 to 2024. The data show that the annual scholarly output has demonstrated a steady increasing trend since 2015, reaching a peak in 2022 (65 articles). This growth pattern reflects the growing recognition of the importance of psychological resilience after stroke (Feigin et al., 2021). As the significance of resilience in stroke rehabilitation has been increasingly validated, related research has evolved from early exploration into an active research domain (Liew et al., 2023). The citation analysis in Figure 2B further supports this developmental trend, showing significant growth in the field’s scholarly impact, with annual citation frequency reaching 1,045 in 2021, and 212 citations already recorded in the first 4 months of 2024, suggesting a sustained high citation rate throughout the year. These findings indicate that research on psychological resilience in stroke survivors has become a continuous scholarly attention, with its scientific value and clinical significance widely acknowledged by researchers (Tsai et al., 2023; Love et al., 2022).

With the continuous expansion of research on resilience in stroke survivors, this study reveals the distribution of scientific research and their significant impact on disciplinary development through analysis of publication volumes by countries, institutions, and authors (Figure 4A). Country analysis results (Figure 4B, Table 2) show that the United States leads the field with 157 publications and the highest centrality (0.38), followed by China (61 publications) and the United Kingdom (33 publications). This distribution pattern is significant for understanding global research development trends in resilience among stroke survivors, revealing the dominant role of developed countries in this field while also demonstrating the research potential of developing countries such as China, providing data support for researchers selecting international collaborators (Muñoz-Venturelli et al., 2022). Meanwhile, through institutional analysis (Figure 4C, Table 3), this study identifies key research institutions such as Harvard University (27 publications), the University of California system (22 publications), and Massachusetts General Hospital (13 publications), providing clear opportunities for academic exchange and collaboration. Author collaboration network analysis (Figures 3A,B) reveals a limited connections between different researchers. While this pattern might partially reflect methodological factors such as variations in author name formats or institutional naming in the database, it likely indicates opportunities for enhanced collaboration in the field of stroke resilience research. This finding suggests the need for strengthening future interdisciplinary and inter-institutional cooperation. Meanwhile, co-cited author analysis (Figure 3C, Table 1) identifies core academic influencers such as Connor KM and Feigin VL. These multi-level analytical results collectively reveal the global scientific research distribution pattern in resilience research among stroke survivors, providing an empirical foundation for optimizing resource allocation and international collaboration in the field of psychological resilience research in stroke (Muñoz-Venturelli et al., 2022).

While analyzing the global distribution of research efforts, this study systematically examined journals and references, revealing the knowledge dissemination network and theoretical development trends in resilience research among stroke survivors, providing new perspectives for understanding the disciplinary structure. The journal analysis (Table 4) identifies Disability and Rehabilitation as the leading journal in terms of publication volume, with nine articles. More importantly, the overall distribution indicates that rehabilitation medicine, neuroscience, and other multidisciplinary journals collectively form the knowledge dissemination system in this field. Co-cited journal analysis (Table 5) further confirms this multidisciplinary characteristic, with “Stroke” (204 citations), “PLoS ONE” (155 citations), and “Proceedings of the National Academy of Sciences” (120 citations) ranking at the top, indicating that stroke resilience research draws on knowledge foundations from both specialized neurology and comprehensive scientific journals. This journal pattern reflects the interdisciplinary nature of psychological resilience research in stroke, suggesting that stroke resilience research requires the integration of multidisciplinary perspectives, establishing a foundation for promoting more comprehensive theoretical construction and intervention strategy development (Chen et al., 2023; Dong et al., 2022).

Dual-map overlay analysis (Figure 5) further shows that literature in neuroscience is significantly influenced by psychology, education, and sociology (*z* = 3.08, *f* = 6,489) as well as health sciences, nursing, and medicine (*z* = 1.97, *f* = 4,396). This finding confirms the important role of multidisciplinary integration in stroke resilience research, providing directional references for guiding future interdisciplinary collaborations (Kanwal et al., 2022; Lo et al., 2023). Reference co-citation analysis (Figure 6, Tables 6, 7) reveals the core theoretical sources in this field, with Connor and Davidson’s assessment tool research (35 citations) and Luthar et al.’s research (18 citations) emerging as the most influential references. The identification of these highly co-cited reference plays an important role in deepening stroke resilience research: on one hand, it clarifies the most recognized theoretical tools and conceptual frameworks in existing research (Connor and Davidson, 2003); on the other hand, it provides guidance for future research directions, helping to promote the field’s transition from conceptual exploration to evidence-based practice. Overall, these bibliometric analysis results systematically demonstrate the knowledge structure of resilience research in stroke survivors, providing empirical support for promoting theoretical development and clinical applications in this field (Qureshi et al., 2023).

### 4.2 Research hotspots in psychological resilience research among stroke survivors

To explore research hotspots in resilience among stroke survivors, this study conducted a systematic analysis of keywords. The keyword co-occurrence network map (Figure 7A) and density visualization map (Figure 7B) intuitively display the research hotspots and thematic distribution in this field, with results showing that core keywords such as “stroke,” “resilience,” “depression,” “health,” and “quality of life” represent the research hotspots in this area. This keyword distribution reflects an important shift in research focus from mere disease management to quality of life enhancement.

This study further revealed the temporal dynamic changes in research hotspots through strongest citation bursts (Table 8). During the period 2014–2019, the emergence of “cognitive impairment” (strength = 2.57) and “risk factors” (strength = 2.49) reflected early research focus on cognitive dysfunction and risk factors, providing references for understanding the fundamental influencing factors of psychological resilience. As research deepened, “resilience” showed significant emergence during 2019–2022 (strength = 2.97), marking it as a core concept in research. The concurrent emergence of “hospital anxiety” (strength = 2.79) and “rehabilitation” (strength = 2.11) indicated that research began to systematically explore the application value of psychological resilience in the stroke rehabilitation process. The emergence of “impact” (strength = 3.02) and “scale” (strength = 2.55) reflected research efforts to quantify the influence of psychological resilience and develop effective assessment tools, which is crucial for establishing empirical connections between psychological resilience and rehabilitation outcomes.

A significant characteristic of recent research hotspots is the development toward clinical application and individualization. The high burst strength of the “people” keyword (strength = 3.54) indicates that research has begun to emphasize a person-centered approach, with greater attention to individual differences and patient needs (Cao et al., 2022). Meanwhile, the emergence of “guidelines” (strength = 2.92) and “symptoms” (strength = 2.19) reflects growing research demands for developing standardized practice guidelines and precise symptom management (Lynch et al., 2022). These latest hotspots suggest that resilience research in stroke survivors is evolving from conceptual and theoretical levels toward practical, individualized, and standardized directions, providing important guidance for the application of psychological resilience in clinical stroke practice (Cormican et al., 2023).

### 4.3 Clinical implications of psychological resilience for stroke survivors

The hotspot analysis has important guiding significance for the clinical application of psychological resilience in stroke. First, resilience assessment should become an essential component of psychological evaluation for stroke survivors (Yan and Lin, 2022). Current research hotspots have expanded from simply focusing on emotional symptoms such as depression and anxiety to the comprehensive assessment of psychological resilience and quality of life, suggesting that clinical practice requires a more comprehensive evaluation system. Regarding the common cognitive impairment in stroke patients, assessment tools should be appropriately adapted to ensure applicability for patients with different functional levels.

Second, resilience cultivation should be integrated into rehabilitation therapy. Research hotspots indicate an increasingly trend of combining rehabilitation and psychological resilience, which aligns with the need for clinical resource optimization and improved patient experience (Wang et al., 2024). In practice, progressive goal setting can be incorporated into physical therapy to enhance patients’ experiences of success. This intervention approach can promote the recovery of physical function and psychological adaptability while improving rehabilitation efficiency without significantly increasing treatment complexity (Norvang et al., 2022).

Finally, clinical implementation of psychological resilience should consider both individualization and standardization. Research hotspots indicate that attention to individual patient characteristics and development of standardized guidelines have become current research trends (Wang et al., 2024). In clinical practice, intervention protocols should be personalized according to patients’ age, cultural background, disease severity, and social support status to meet the specific needs of different patients (Yan and Lin, 2022). Meanwhile, the development of standardized guidelines helps ensure intervention quality. These guidelines should clearly specify applicable populations, intervention timing, core content, and assessment methods, providing theoretical guidance for clinical practice (Qureshi et al., 2023). Through the combination of individualized implementation and standardized principles, psychological resilience interventions can be more effectively integrated into stroke rehabilitation systems, enhancing patients’ psychological adaptability and quality of life.

### 4.4 Study strengths and limitations

This study employed bibliometric analysis to analyze 424 publications on resilience in stroke survivors published between 2000 and 2024, providing a comprehensive understanding of the current research trends in this field. Through the application of visualization tools such as VOSviewer and CiteSpace, we conducted multi-dimensional analyses of authors, countries, institutions, journals, references, and keywords, presenting research hotspots and frontier dynamics. This study not only constructed the knowledge map of this field but also focused on the clinical application value of psychological resilience in stroke survivors, providing evidence-based support for optimizing clinical intervention strategies. This systematic bibliometric analysis also provides important references for future research directions.

However, this study has several limitations. First, due to software technical constraints, we only included English publications from the WoSCC database, potentially excluding high-quality research published in other languages. Second, this study did not differentiate between ischemic stroke and hemorrhagic stroke subtypes, while differences in these pathological mechanisms may lead to different psychological responses in patients, affecting the precision of results. Third, in the author collaboration network, we observed that low centrality values may be partially attributed to inconsistent spelling of author names, an issue difficult to resolve in the current study due to the lack of unified identifiers (such as ORCID) in the original database and the complexity of post-processing standardization techniques. Finally, when evaluating institutional influence, we were unable to distinguish between “high-productivity institutions” and “high-impact institutions,” primarily limited by technical challenges in standardizing institution names during data extraction, making it unfeasible to calculate citation frequencies for institutions.

Despite these limitations, this study still provides a systematic perspective for understanding the current status and future directions in the field of resilience among stroke survivors. Future studies may consider integrating multi-database resources, including literature in a wider range of languages, and combining qualitative content analysis to gain a more comprehensive and in-depth understanding of this field.

## 5 Conclusion

This bibliometric analysis examined 424 articles on resilience in stroke survivors published between 2000 and 2024. The analysis identified Zhang W and Vranceanu A as the most productive authors. The United States, China, and the United Kingdom emerged as the primary contributing countries, with Harvard University publishing 27 articles as the most productive institution. Journal analysis revealed that “Disability and Rehabilitation” and “Stroke” played significant roles in this field. Knowledge structure analysis demonstrated a shift in research focus from disease-related factors toward resilience assessment and intervention. This shift not only reflects the developmental progress in resilience research but also provides important evidence for improving rehabilitation outcomes and quality of life for stroke survivors. Future research should address psychological resilience characteristics in patients with different stroke subtypes, develop targeted assessment tools and intervention strategies, and promote the effective application of research findings in clinical practice.

## Data Availability

The original contributions presented in this study are included in this article/supplementary material, further inquiries can be directed to the corresponding author.

## References

[B1] Bin Suliman M. A. Hanis T. M. Kamdi M. K. A. Ibrahim M. I. Musa K. I. (2023). A bibliometric analysis of stroke caregiver research from 1989 to 2022. *Int. J. Environ. Res. Public Health* 20:4642. 10.3390/ijerph20054642 36901652 PMC10001807

[B2] Cao L. L. Tang Y. F. Xia Y. Q. Wei J. H. Li G. R. Mu X. M. (2022). A survey of caregiver burden for stroke survivors in non-teaching hospitals in Western China. *Medicine* 101:e31153. 10.1097/MD.000000000003a1153 36550813 PMC9771191

[B3] Chen M. Zhang Y. Dong L. Guo X. (2023). Bibliometric analysis of stroke and quality of life. *Front. Neurol.* 14:1143713. 10.3389/fneur.2023.1143713 37114223 PMC10128914

[B4] Connor K. M. Davidson J. R. (2003). Development of a new resilience scale: The connor-davidson resilience scale (CD-RISC). *Depression Anxiety* 18 76–82. 10.1002/da.10113 12964174

[B5] Cormican A. Hirani S. P. McKeown E. (2023). Healthcare professionals’ perceived barriers and facilitators of implementing clinical practice guidelines for stroke rehabilitation: A systematic review. *Clin. Rehabil.* 37 701–712. 10.1177/02692155221141036 36475911 PMC10041573

[B6] Dong Y. Weng L. Hu Y. Mao Y. Zhang Y. Lu Z. (2022). Exercise for stroke rehabilitation: A bibliometric analysis of global research from 2001 to 2021. *Front. Aging Neurosci.* 14:876954. 10.3389/fnagi.2022.876954 35783146 PMC9247282

[B7] Donthu N. Kumar S. Mukherjee D. Pandey N. Lim W. M. (2021). How to conduct a bibliometric analysis: An overview and guidelines. *J. Business Res.* 133 285–296. 10.1016/j.jbusres.2021.04.070

[B8] Fan F. Lei M. (2022). Mechanisms underlying curcumin-induced neuroprotection in cerebral ischemia. *Front. Pharmacol.* 13:893118. 10.3389/fphar.2022.893118 35559238 PMC9090137

[B9] Feigin V. L. Stark B. A. Johnson C. O. Roth G. A. Bisignano C. Abady G. G. (2021). Global, regional, and national burden of stroke and its risk factors, 1990–2019: A systematic analysis for the Global Burden of Disease Study 2019. *Lancet Neurol.* 20 795–820. 10.1016/S1474-4422(21)00252-0 34487721 PMC8443449

[B10] Gugliandolo A. Silvestro S. Sindona C. Bramanti P. Mazzon E. (2021). MiRNA: Involvement of the MAPK pathway in ischemic stroke. A promising therapeutic target. *Medicina* 57:1053. 10.3390/medicina57101053 34684090 PMC8539390

[B11] Han Z. Zhang H. Wang Y. Zhu S. Wang D. (2021). Uncertainty in illness and coping styles: Moderating and mediating effects of resilience in stroke patients. *World J. Clin. Cases* 9:8999. 10.12998/wjcc.v9.i30.8999 34786383 PMC8567502

[B12] Heltty H. Zahalim Z. (2023). Resilience after stroke and its correlation with functional independence. *J. Ners.* 18 57–63. 10.20473/jn.v18i1

[B13] Kanwal S. Hashmi R. Perveen W. Ali M. A. Akhtar M. Munawar A. (2022). Outcomes of multidisciplinary team approach in rehabilitation of patients with stroke. *Pak. J. Med. Health Sci.* 16 78–78. 10.53350/pjmhs2216378

[B14] Liew S. L. Schweighofer N. Cole J. H. Zavaliangos-Petropulu A. Lo B. P. Han L. K. (2023). Association of brain age, lesion volume, and functional outcome in patients with stroke. *Neurology* 100 e2103–e2113. 10.1212/WNL.0000000000202113 37015818 PMC10186236

[B15] Liu W. Huang M. Wang H. (2021). Same journal but different numbers of published records indexed in scopus and web of science core collection: Causes, consequences, and solutions. *Scientometrics* 126 4541–4550. 10.1007/s11192-021-04034-0

[B16] Lo S. H. S. Chau J. P. C. Lau A. Y. L. Choi K. C. Shum E. W. C. Lee V. W. Y. (2023). Virtual multidisciplinary stroke care clinic for community-dwelling stroke survivors: A randomized controlled trial. *Stroke* 54 2482–2490. 10.1161/STROKEAHA.123.043566 37551588 PMC10519295

[B17] Love M. F. Brooks A. N. Cox S. D. Okpala M. Cooksey G. Cohen A. S. (2022). The effects of racism and resilience on Black stroke-survivor quality of life: Study protocol and rationale for a mixed-methods approach. *Front. Neurol.* 13:885374. 10.3389/fneur.2022.885374 36034272 PMC9399920

[B18] Lynch E. A. Connell L. A. Carvalho L. B. Bird M. L. (2022). Do clinical guidelines guide clinical practice in stroke rehabilitation? An international survey of health professionals. *Disability Rehabil.* 44 4118–4125. 10.1080/09638288.2021.1915673 33651965

[B19] Markscheffel B. Schröter F. (2021). Comparison of two science mapping tools based on software technical evaluation and bibliometric case studies. *Collnet J. Scientometrics Information Manag.* 15 365–396. 10.1080/09737766.2021.1924477

[B20] Muñoz-Venturelli P. González F. Urrutia F. Mazzon E. Navia V. Brunser A. (2022). Stroke care and collaborative academic research in Latin America. *Salud Pública de México* 64 S40–S45. 10.21149/12803 36130397

[B21] Muraleedharan M. Chandak A. O. (2023). Global insights: A bibliometric analysis of research trends in stroke thrombolysis. *J. Stroke Med.* 6 83–88. 10.1177/25166085231200766

[B22] Nikolovska L. Taci A. (2023). Physical therapy and neurorehabilitation of patients with cerebrovascular stroke. *Medis Int. J. Med. Sci. Res.* 2 61–65. 10.35120/medisij020461n 27599931

[B23] Norvang O. P. Dahl A. E. Thingstad P. Askim T. (2022). Resilience and its association with activities of daily living 3 months after stroke. *Front. Neurol.* 13:881621. 10.3389/fneur.2022.881621 35775055 PMC9237386

[B24] Ohashi S. N. DeLong J. H. Kozberg M. G. Mazur-Hart D. J. Van Veluw S. J. Alkayed N. J. (2023). Role of inflammatory processes in hemorrhagic stroke. *Stroke* 54 605–619. 10.1161/STROKEAHA.122.037155 36601948

[B25] Qureshi A. Hargest C. Swain N. Aldabe D. Hale L. (2023). Psychosocial interventions for building resilience of informal carers of people living with stroke: A systematic review. *Disability Rehabil.* 45 1419–1432. 10.1080/09638288.2022.2063419 35468030

[B26] Sirdani M. Zohreh-Vand F. Torabi M. (2021). Stroke as a neurodegenerative disease; A review of the introduction, epidemiology, diagnosis, complications and causes. *Central Asian J. Med. Pharm. Sci. Innov.* 1 156–164. 10.22034/CAJMPSI.2021.03.06 36190387

[B27] Stuckey S. M. Ong L. K. Collins-Praino L. E. Turner R. J. (2021). Neuroinflammation as a key driver of secondary neurodegeneration following stroke? *Int. J. Mol. Sci.* 22:13101. 10.3390/ijms222313101 34884906 PMC8658328

[B28] Tsai S. J. Li C. C. Tsai S. M. Kao S. C. Pai H. C. (2023). The effect of action modules on resilience and psychological health of stroke patients: A pilot non-randomised control trial. *J. Clin. Nurs.* 32 311–319. 10.1111/jocn.16295 35118746

[B29] Wang Y. Xie H. Sun H. Ren L. Jiang H. Chen M. (2024). Influencing factors of psychological resilience in stroke patients: A systematic review and meta-analysis. *Arch. Clin. Neuropsychol.* 39 644–654. 10.1093/arclin/acad107 38324660

[B30] Xiong F. Liao X. Xiao J. Bai X. Huang J. Zhang B. (2022). Emerging limb rehabilitation therapy after post-stroke motor recovery. *Front. Aging Neurosci.* 14:863379. 10.3389/fnagi.2022.863379 35401147 PMC8984121

[B31] Yan H. Y. Lin H. R. (2022). Resilience in stroke patients: A concept analysis. *Healthcare* 10:2281. 10.3390/healthcare10112281 36421605 PMC9691242

[B32] Yan L. Zhiping W. (2023). Mapping the literature on academic publishing: A bibliometric analysis on WOS. *Sage Open* 13:21582440231158562. 10.1177/21582440231158562

[B33] Zafar L. Masood N. (2020). Impact of field of study trend on scientific articles. *IEEE Access* 8 128295–128307. 10.1109/ACCESS.2020.3007558

[B34] Zhang D. Ding W. Wang Y. Liu S. (2022). Exploring the role of international research collaboration in building China’s world-class universities. *Sustainability* 14:3487. 10.3390/su14063487

